# Water vapor oxidation behavior of high-entropy (Ti,Nb,Ta,Hf,W)C_*x*_ at 1200 °C: the role of carbon vacancies

**DOI:** 10.1039/d6ra04394a

**Published:** 2026-08-17

**Authors:** Wei Liao, Li Xu, Jiashan Xie, Xiaosong Zhou, Yongqiang Tan

**Affiliations:** a Department of Materials Science and Engineering, University of Science and Technology of China Hefei 230026 China; b Institute of Nuclear Physics and Chemistry, China Academy of Engineering Physics Mianyang 621900 China

## Abstract

The oxidation behavior of (Ti,Nb,Ta,Hf,W)C_*x*_ in high-temperature water vapor was studied. The results indicate that the replacement of Zr with W enhances the oxidation resistance of high-entropy (Ti,Zr,Nb,Ta,Hf)C. Moreover, with increasing carbon vacancies, the oxidation resistance of (Ti,Nb,Ta,Hf,W)C_*x*_ further increases, and (Ti,Nb,Ta,Hf,W)C_0.8_ displays optimal oxidation resistance. The weaker O atom adsorbing ability and the lower diffusion ability of W are among the main reasons for the superior oxidation resistance. Additionally, the decrease in residual carbon in the oxidation layer with increasing carbon vacancies and the disappearance of the (Nb,Ta)_2_O_5_ phase in the oxide scale on reaction with WO_2_ effectively inhibit the inward diffusion of water vapor.

## Abstract 2

Synergistic enhancement of water vapor oxidation resistance.

## Graphical abstract

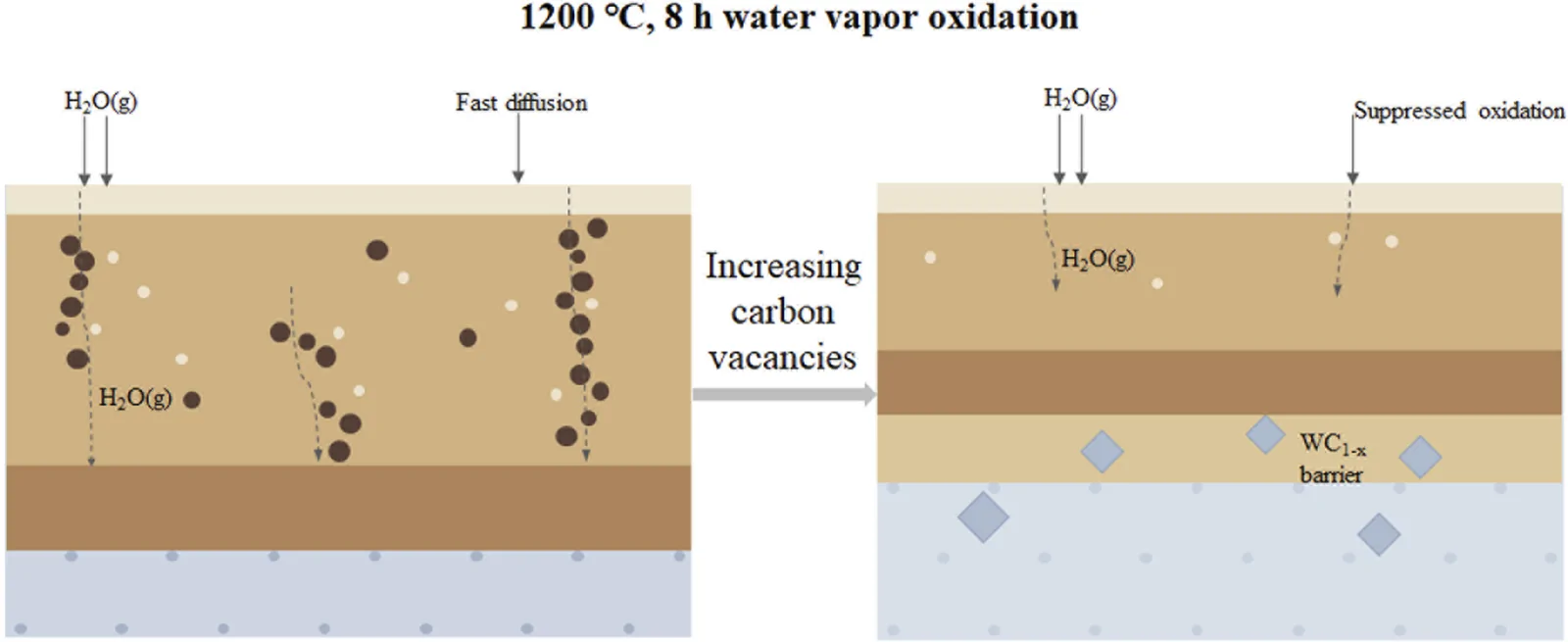

## Introduction

Transition-metal carbides (TMCs), such as ZrC and TiC, possess a unique combination of ultra-high melting points (exceeding 3000 °C), high thermal conductivity, and outstanding high-temperature mechanical strength.^1,2^ These exceptional properties make them indispensable materials for structural components operating in extreme thermal environments, including leading edges and thermal protection systems (TPS) for hypersonic aerospace vehicles, throats of rocket propulsion nozzles, and accident-tolerant fuel claddings in advanced nuclear reactors.^3–5^ However, most transition-metal carbides suffer from poor oxidation resistance, which is the main obstacle for the viability of carbides in high-temperature environments.^6–9^ Recently, high-entropy transition-metal carbides (HECs) were discovered to show many interesting properties, one of which is the enhanced oxidation resistance in both air and water steam environments compared to their corresponding monocarbides.^10,11^ More importantly, recent studies have demonstrated that the oxidation resistance temperature of HECs can be elevated to an unprecedented 3600 °C through a high-entropy compositional engineering strategy, far exceeding the limits of conventional ultra-high temperature ceramics.^12^ Generally, the oxidation behavior of most HECs followed a parabolic rate law, indicating a diffusion-controlled oxidation process and good protection against further oxidation by the dense mixed-oxide layers.^13–19^ In our previous studies, it was demonstrated that the weight gain of (Zr,Ti,Nb,Ta,Hf)C is almost one magnitude lower than that of ZrC in water vapor at 1200 °C.^17^ Even for powders, high-entropy compositions display better oxidation resistance. Wang *et al.* compared the oxidation behaviours of (Hf,Ta,Zr,Nb)C powder and the corresponding monocarbide (HfC, TaC, ZrC, and NbC) powders, and the results indicated that (Hf,Ta,Zr,Nb)C powder has a higher oxidation onset temperature than its four component monocarbides, and it maintained the original shape after oxidation.^20^

It was predicted and then experimentally verified that the oxidation of high-entropy materials occurs by preferential oxidation of each component according to the relative thermodynamic favorabilities of their respective oxides.^21,22^ Based on preferential oxidation, a compositional design strategy involving reduction of elements with higher oxygen adsorption energies, such as Nb, Ta, and Ti, in HECs was recently developed by first-principles calculations.^16^ The oxidation performance of HECs was effectively improved by regulating preferential oxidation and suppressing the formation of detrimental oxides during the oxidation process.^16^ This is highly consistent with our previous studies, which showed that the oxidation behaviour of HECs shows a clear compositional dependence.^19,23^

Binary WC, which possesses excellent mechanical property and high flexural strength, was reported to form within (Ti,Zr,Hf,Nb,Ta,W_*x*_)C.^24^ However, most W-containing compositions demonstrate relatively lower entropy-forming ability.^25^ Recently, high-entropy compositions containing W have received growing interest, and several single-phase high-entropy compositions with excellent mechanical properties and oxidation resistance have been reported.^24,26–30^ The antioxidation properties of HECs containing W were discovered to be affected by the W content due to its high resistance to oxygen absorption.^27^ Specifically, Chu *et al.* reported a novel high-entropy carbide (Hf,Ta,Zr,W)C with exceptional oxidation resistance up to 3600 °C, wherein the superior oxidation resistance of W atoms plays an important role.^31^

In our previous study, it was discovered that the phase formation and mechanical properties of high-entropy (Ti_0.2_Nb_0.2_Ta_0.2_Hf_0.2_W_0.2_)C_*x*_ vary with carbon vacancies.^26^ However, the influence of carbon vacancies on the oxidation resistance of (Ti_0.2_Nb_0.2_Ta_0.2_Hf_0.2_W_0.2_)C_*x*_ is still unclear. Besides, (Ti_0.2_Nb_0.2_Ta_0.2_Hf_0.2_W_0.2_)C_*x*_ contains elements with greatly different diffusion ability, and a systematic study of their oxidation behaviour will help to gain a deeper understanding of the oxidation mechanisms of high-entropy carbides.

## Experimental method

(Ti_0.2_Nb_0.2_Ta_0.2_Hf_0.2_W_0.2_)C_*x*_ (*x* = 1.0, 0.9, 0.8) with different carbon vacancies were prepared by spark plasma sintering (SPS) of TiC (Aladdin, 99% purity, 2–4 µm powder size), NbC (Aladdin, 99% purity, 1–4 µm powder size), TaC (Changyu, 99.5% purity, ≤500 nm powder size), HfC (Aladdin, 99.5% purity, 2–4 µm powder size), WC (Aladdin, 99% purity, ≤1 µm powder size) and TiH_2_ (Forsman, 99.5% purity, ≤45 µm powder size). Details of the preparation were provided in our previous study.^26^ The as-prepared (Ti_0.2_Nb_0.2_Ta_0.2_Hf_0.2_W_0.2_)C_*x*_ bulk materials exhibit nearly full densification. The Archimedes method measured densities of 10.92 g cm^−3^, 11.04 g cm^−3^, and 11.14 g cm^−3^, and open porosities of 2.6%, 1.5%, and 0.1% for *x* = 1.0, 0.9, and 0.8, respectively. The phase identification and microstructure were examined by XRD and SEM-EDS, respectively. The results confirm that all samples are single-phase solid solutions, except for the *x* = 0.8 composition, which contains a uniformly distributed rock-salt structured WC_1−*x*_ second phase (consistent with our previous work^26^).

Isothermal oxidation tests were performed on samples with dimensions of 4 mm × 4 mm × 4 mm. All samples were highly polished using a 1 µm diamond paste. The high-temperature water vapor oxidation tests were performed using thermogravimetric analysis (TGA; Setaram, France). An automated wet gas generator, Wetsys (Setaram), regulated the relative humidity (RH) of the TGA chamber, and a humidity level of 90% H_2_O was achieved by passing the experimental gas (Ar) through a water bath. The oxidation temperature was 1200 °C, and the oxidation duration was 8 h. Mass changes were recorded continuously as a function of time during the isothermal oxidation.

The phase information was characterized by X-ray diffractometry (XRD, Bruker AXS Ltd). The microstructures of the specimens after oxidation were observed using scanning electron microscopy (SEM, Zeiss) and energy-dispersive X-ray spectroscopy (EDS).

## Results and discussions

The detailed microstructural characterization of the investigated carbides was reported in our previous study and thus is not shown here for brevity. All the carbide solid solutions show dense microstructures. (Ti_0.2_Nb_0.2_Ta_0.2_Hf_0.2_W_0.2_)C and (Ti_0.2_Nb_0.2_Ta_0.2_Hf_0.2_W_0.2_)C_0.9_ show single phases with uniform elemental distributions, and (Ti_0.2_Nb_0.2_Ta_0.2_Hf_0.2_W_0.2_)C_0.8_ contains some second phase WC_1−*x*_.^26^

**Fig. 1:**
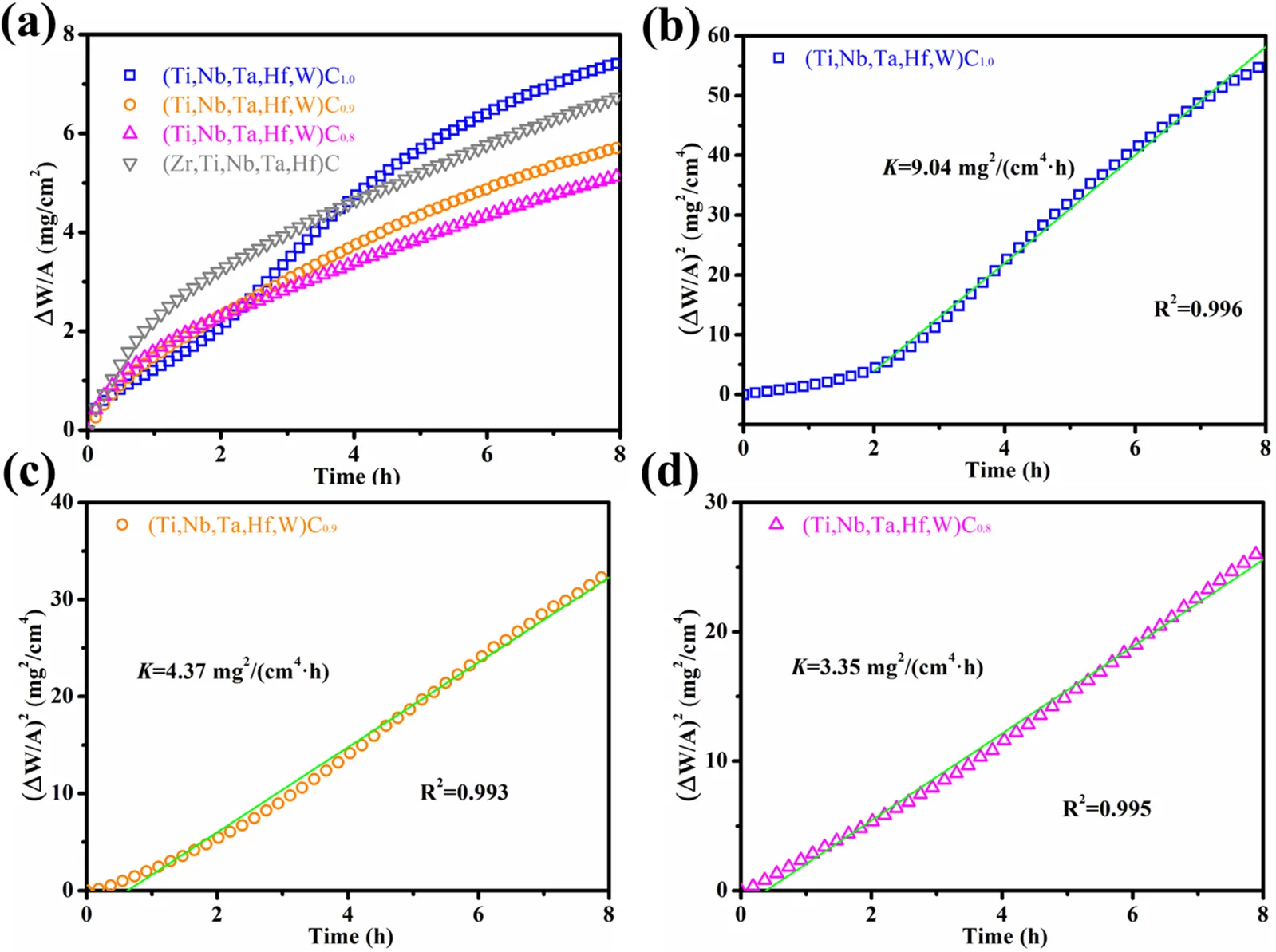
(a) Weight gain per unit area (Δ*W*/*A*) as a function of exposure time for (Ti_0.2_Nb_0.2_Ta_0.2_Hf_0.2_W_0.2_)C_*x*_ with different x and (Zr,Ti,Nb,Ta,Hf)C, (b) the kinetic fitting of oxidation data for (Ti_0.2_Nb_0.2_Ta_0.2_Hf_0.2_W_0.2_)C using the parobolic rate law, and the kinetic fitting of oxidation data for (c) (Ti_0.2_Nb_0.2_Ta_0.2_Hf_0.2_W_0.2_)C_0.9_ and (d) (Ti_0.2_Nb_0.2_Ta_0.2_Hf_0.2_W_0.2_)C_0.8_ using a power rate equation.

Fig. 1 compares the weight gain per unit area (Δ*W*/*A*) as a function of exposure time for (Ti_0.2_Nb_0.2_Ta_0.2_Hf_0.2_W_0.2_)C_*x*_ with different *x*. The Δ*W*/*A*–*t* curves were fitted with a power rate equation as follows,1
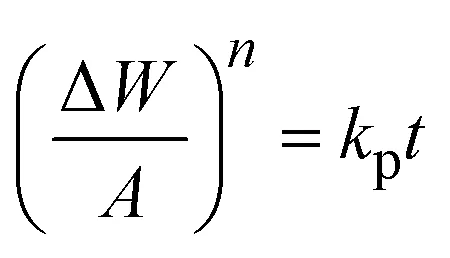
where *k*_p_ is a constant related to the diffusion coefficient. For (Ti_0.2_Nb_0.2_Ta_0.2_Hf_0.2_W_0.2_)C, the weight gain curve can be divided into two stages. Both stages can be fitted with the parabolic rate law; however, the second stage (*t* ≥ 2 h) shows a much higher *k*_p_, which suggests accelerated oxidation after 2 h. During the first stage (*t* ≤ 2.5 h), (Ti_0.2_Nb_0.2_Ta_0.2_Hf_0.2_W_0.2_)C shows the lowest weight gain; with increasing carbon vacancies the weight gain gradually increases, which is consistent with the oxidation of (Zr,Ti,Nb,Ta,Hf)C_0.8_.^32^ However, during the second stage (Ti_0.2_Nb_0.2_Ta_0.2_Hf_0.2_W_0.2_)C shows the largest weight gain, followed by (Ti_0.2_Nb_0.2_Ta_0.2_Hf_0.2_W_0.2_)C_0.9_, and (Ti_0.2_Nb_0.2_Ta_0.2_Hf_0.2_W_0.2_)C_0.8_ shows the smallest weight gain of only 5.1 mg cm^−2^ after oxidation for 8 h, which is much smaller than that of (Zr,Ti,Nb,Ta,Hf)C under the same oxidation condition (6.8 mg cm^−2^). Besides, the oxidation curves of both (Ti_0.2_Nb_0.2_Ta_0.2_Hf_0.2_W_0.2_)C_0.9_ and (Ti_0.2_Nb_0.2_Ta_0.2_Hf_0.2_W_0.2_)C_0.8_ can be fitted by a power rate equation with an exponent *n* = 2 during the whole stage.

**Fig. 2:**
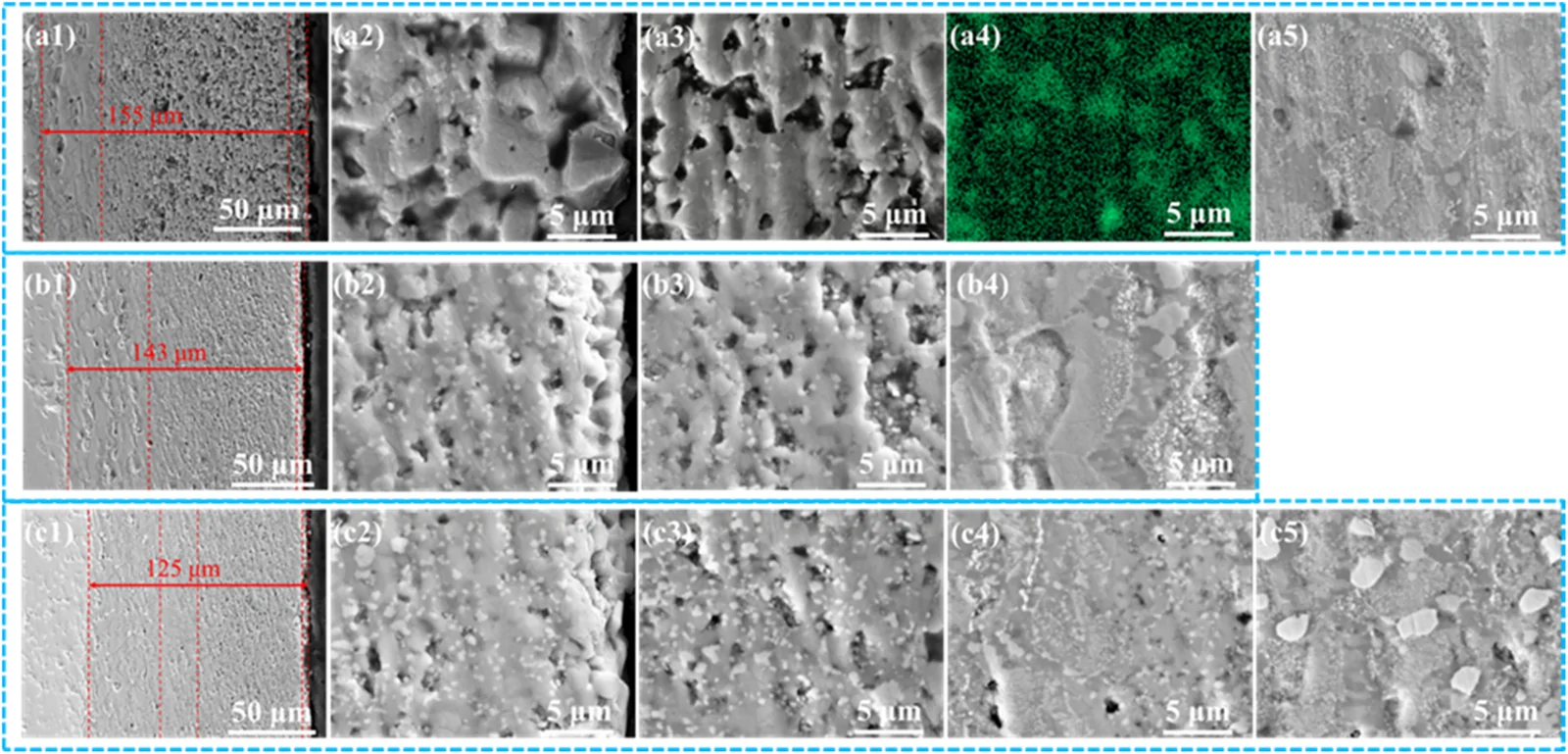
Cross-sectional SEM morphologies of the oxidation layers of (a1)–(a5) (Ti_0.2_Nb_0.2_Ta_0.2_Hf_0.2_W_0.2_)C; (b1)–(b4) (Ti_0.2_Nb_0.2_Ta_0.2_Hf_0.2_W_0.2_)C_0.9_; and (c1)–(c5) (Ti_0.2_Nb_0.2_Ta_0.2_Hf_0.2_W_0.2_)C_0.8_ after oxidation. Here, (a4) is the corresponding EDS spectrum of the carbon element for (a3).

Fig. 2 shows the cross-sectional SEM morphologies of the oxidation layers of (Ti_0.2_Nb_0.2_Ta_0.2_Hf_0.2_W_0.2_)C_*x*_ after oxidation at 1200 °C. All oxidation layers appear to adhere well to the residual carbide without cracking or spalling. A three-layer structure with a fully oxidized thickness of approximately 155 µm is observed in (Ti_0.2_Nb_0.2_Ta_0.2_Hf_0.2_W_0.2_)C. The outer layer is a thin layer of around 10.5 µm with a relatively large grain size (Fig. 2(a2)). The middle layer shows a ∼113.2 µm thickness, and a large amount of residual carbon phase can be distinguished by the EDS mapping of carbon, as shown in Fig. 2(a4). The inner layer with a thickness of 31.1 µm shows a dense microstructure and fine grain size. With increasing carbon vacancies, the thickness of the total oxidation layer decreases, which is consistent with their final weight gain. Besides, the amount of residual carbon phase in the middle layer is much less in (Ti_0.2_Nb_0.2_Ta_0.2_Hf_0.2_W_0.2_)C_0.9_ and (Ti_0.2_Nb_0.2_Ta_0.2_Hf_0.2_W_0.2_)C_0.8_. For (Ti_0.2_Nb_0.2_Ta_0.2_Hf_0.2_W_0.2_)C_0.8_, the total thickness of the oxidation layer is 125 µm. An additional inner layer containing unaffected WC_1−*x*_ grains is observed, and the thickness of the innermost layer is ∼42.5 µm.

**Fig. 3:**
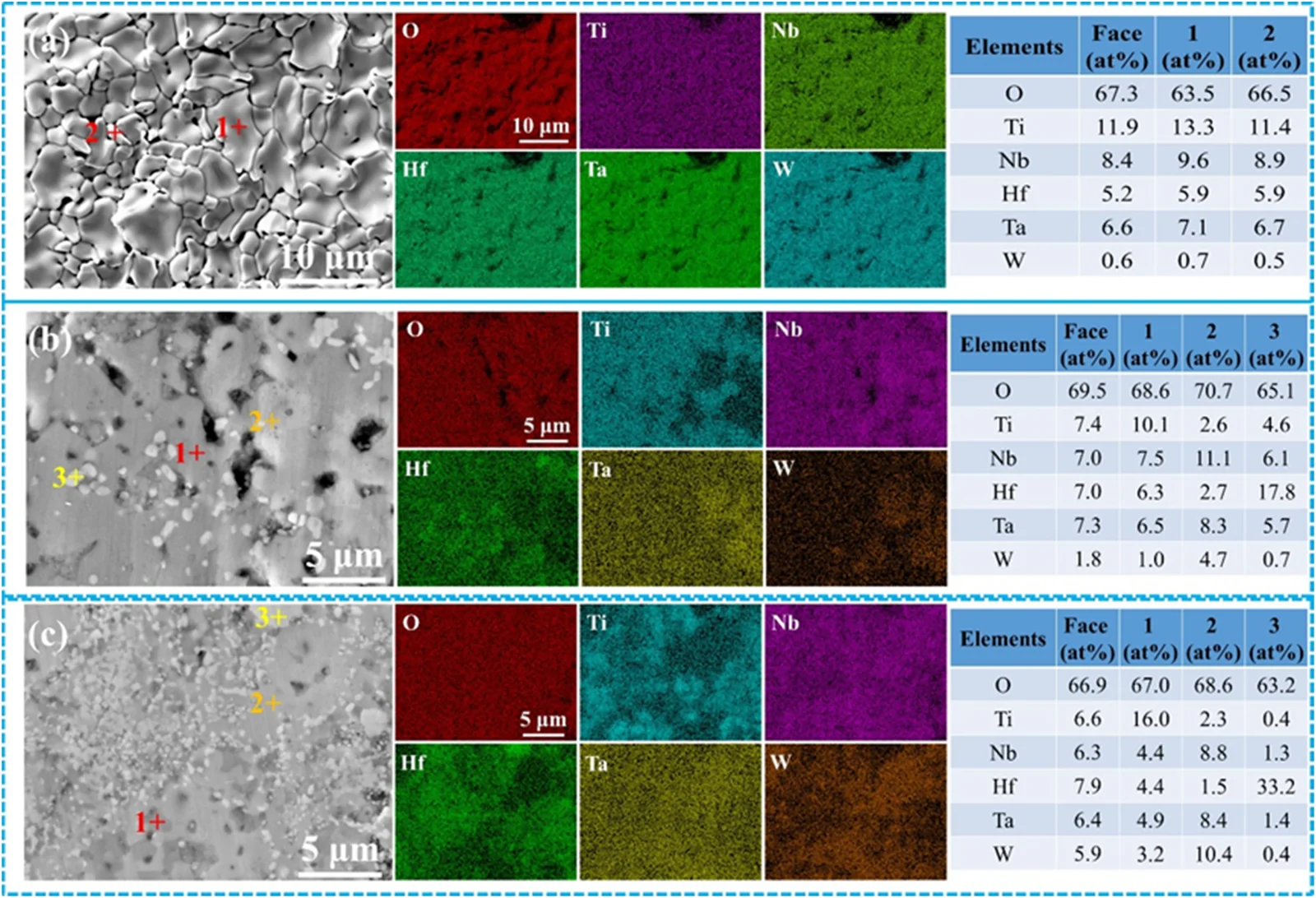
SEM images and corresponding EDS mappings of the three typical layers in the oxide scale of (Ti_0.2_Nb_0.2_Ta_0.2_Hf_0.2_W_0.2_)C (a) outermost layer; (b) second layer after polishing down to a depth of 30 µm from the top surface; and (c) third layer after polishing down to a depth of 130 µm from the top surface.

**Fig. 4:**
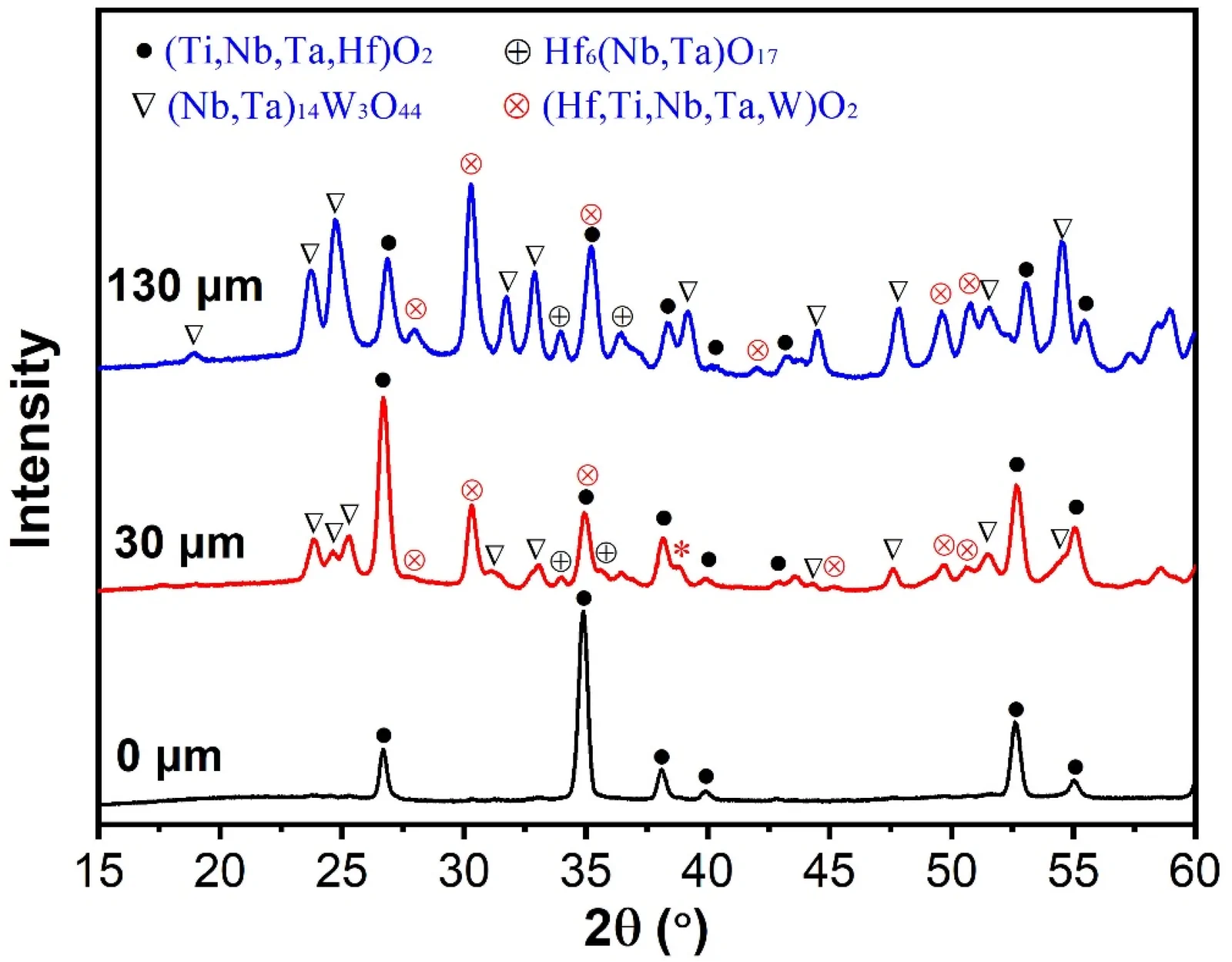
XRD patterns of different layers in the oxide scale of (Ti_0.2_Nb_0.2_Ta_0.2_Hf_0.2_W_0.2_)C.

To characterize the phase composition and elemental distribution across the oxide scale, we performed SEM-EDS and XRD analyses at selected depths within the oxide layer. The selection of these depths was based on the distinct phase regions (*e.g.*, outer layer, middle mixed-oxide layer, and inner layer) observed in the cross-sectional morphology (Fig. 2), rather than a fixed physical thickness, as the total oxide layer thickness varies among the three samples due to their different oxidation resistances. The SEM images and corresponding EDS mappings of the three typical layers in the oxide scale of (Ti_0.2_Nb_0.2_Ta_0.2_Hf_0.2_W_0.2_)C are shown in Fig. 3, and the XRD patterns of the different layers are shown in Fig. 4. The outermost layer shows a porous structure with large grains of approximately 10 µm and smaller grains of approximately 2 µm. EDS mappings indicate that single-phase Ti-rich oxides with extremely low W content were formed at the surface. The XRD patterns also indicate a single-phase rutile TiO_2_ structure, as demonstrated in Fig. 4. Therefore, the outermost layer can be determined to be Nb-, Ta- and Hf-doped TiO_2_. When polished down to a depth of 30 µm from the top surface, from SEM images with EDS mapping, a denser layer with mixed Ti-rich, Nb-rich and Hf-rich oxides can be observed. The total amounts of Ti, Nb, Ta, and Hf are almost the same, with much less W content. Most W formed oxide solid solutions with Nb and Ta. According to the XRD results, the Nb-rich oxides are (Nb,Ta)_14_W_3_O_44_, and the Hf-rich oxides are (Hf,Ti,Nb,Ta,W)O_2_ with a crystal structure as HfO_2_. The XRD patterns corresponding to Hf_6_(Nb,Ta)_2_O_17,_ which is one of the common oxide solid solutions during oxidation of HECs, can be distinguished.^18,27^ Besides, residual carbon can also be distinguished. When further polished down to a depth of 130 µm from the top surface, similar mixed oxides can be observed; however, the W content becomes much higher. Similarly, most W tends to form oxide solid solutions of (Nb,Ta)_14_W_3_O_44_ with Nb and Ta.

**Fig. 5:**
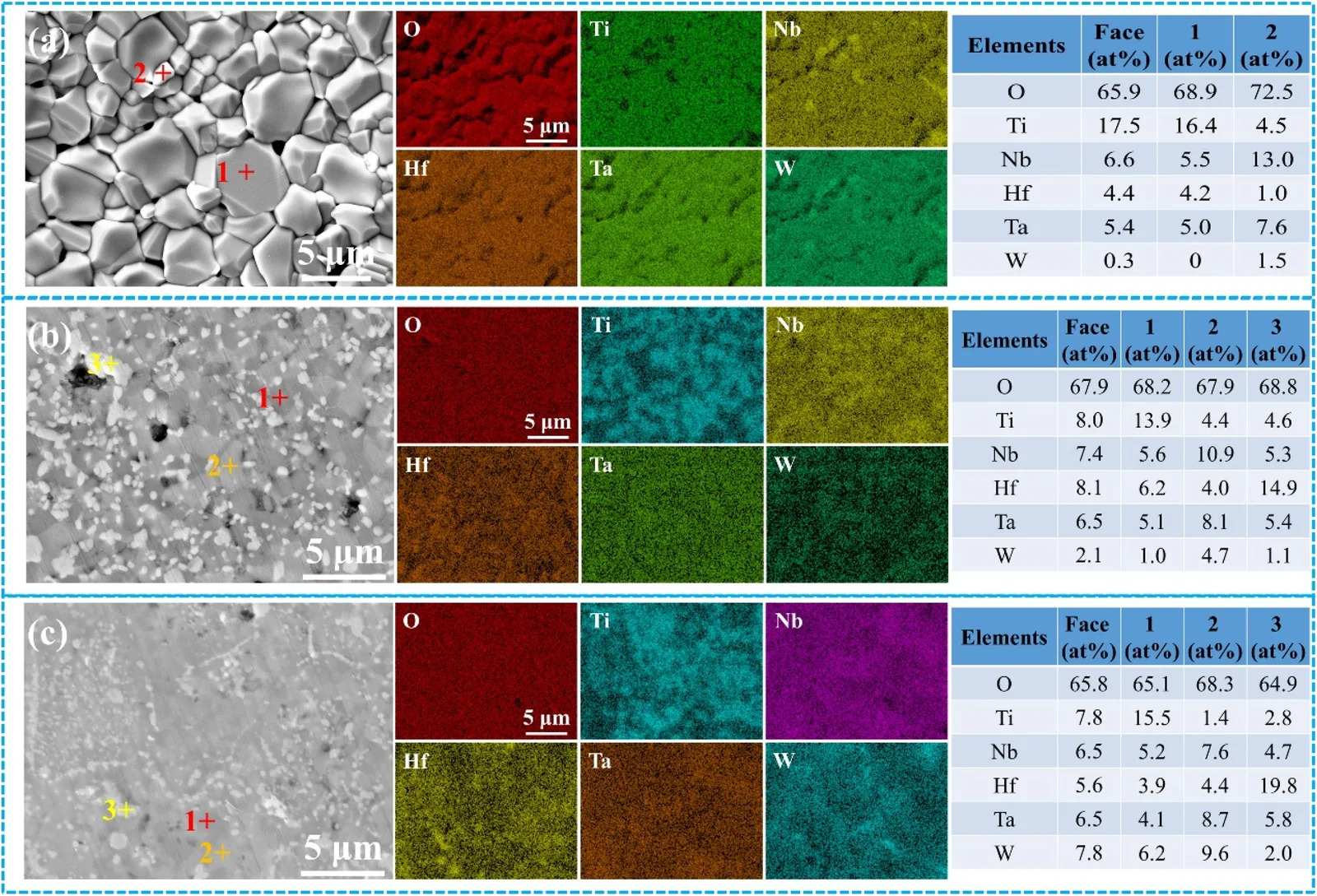
SEM images and corresponding EDS mappings of the three typical layers in the oxide scale of (Ti_0.2_Nb_0.2_Ta_0.2_Hf_0.2_W_0.2_)C_0.9_ (a) outermost layer; (b) second layer after polishing down to a depth of 30 µm from the top surface; and (c) third layer after polishing down to a depth of 100 µm from the top surface.

**Fig. 6:**
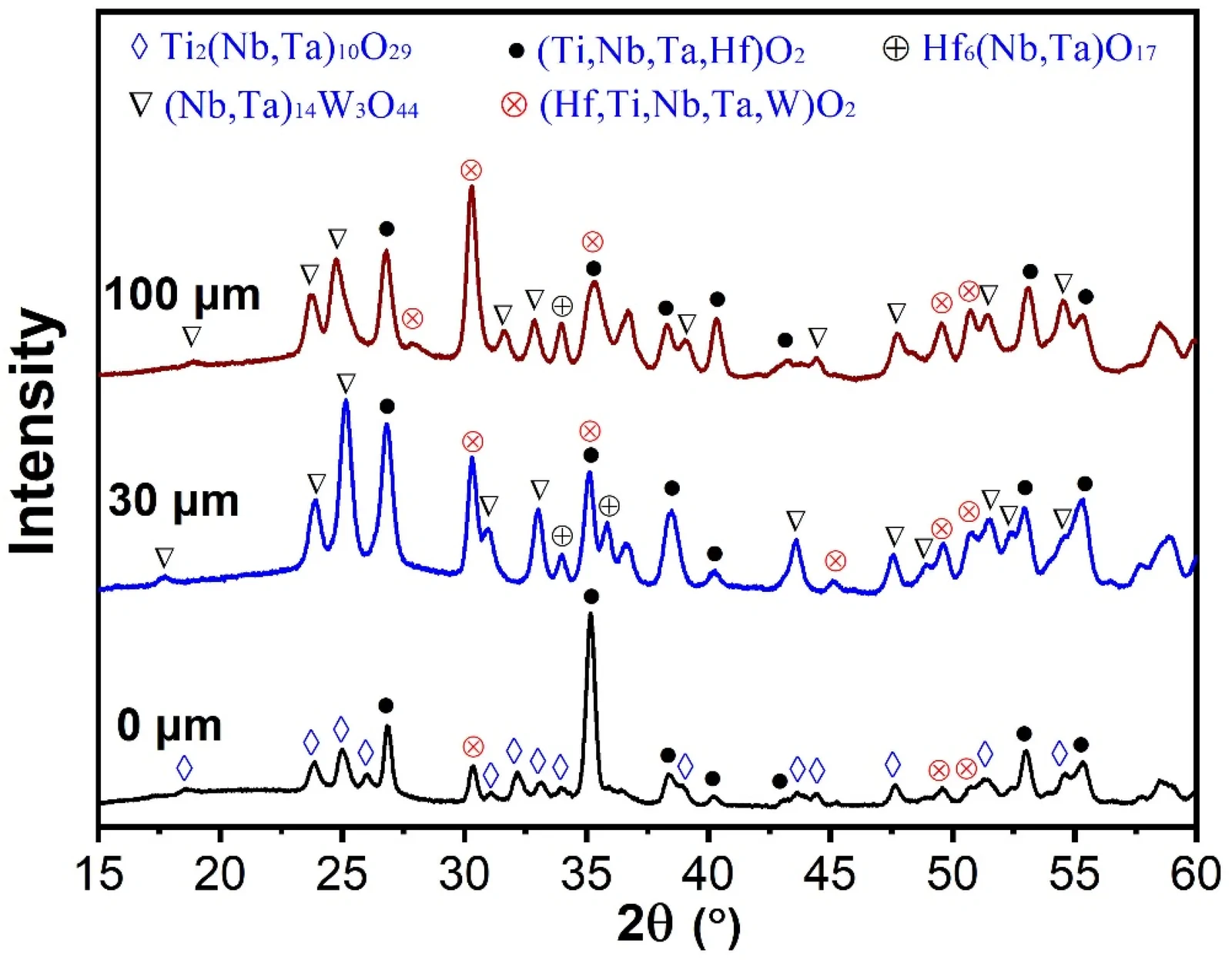
XRD patterns of different layers in the oxide scale of (Ti_0.2_Nb_0.2_Ta_0.2_Hf_0.2_W_0.2_)C_0.9_.

Fig. 5 displays the SEM images and corresponding EDS mappings of the three typical layers in the oxide scale of (Ti_0.2_Nb_0.2_Ta_0.2_Hf_0.2_W_0.2_)C_0.9_. EDS mappings also indicate that Ti-rich oxides (Ti,Nb,Ta,Hf)O_2_ with almost no W are dominant in the outermost surface, and the Ti content is higher compared to that in (Ti_0.2_Nb_0.2_Ta_0.2_Hf_0.2_W_0.2_)C. The Ti-rich oxides show a smaller grain size. Besides, some Nb-rich oxides with even smaller grain size of around 1 µm begin to appear; these are Ti_2_(Nb,Ta)_10_O_29_ according to the XRD patterns shown in Fig. 6. When polished down to a depth of 30 µm from the top surface, a denser layer with mixed Ti-rich (Ti,Nb,Ta,Hf)O_2_, Nb-rich (Nb,Ta)_14_W_3_O_44_ and Hf-rich (Hf,Ti,Nb,Ta,W)O_2_ can be easily distinguished. The Ti-rich (Ti,Nb,Ta,Hf)O_2_ and Nb-rich (Nb,Ta)_14_W_3_O_44_ correspond to the dark grey and light grey grains, respectively, with similar grain sizes of approximately 2 µm. The Hf-rich oxides (Hf,Ti,Nb,Ta,W)O_2_ correspond to the white particles with a grain size smaller than 1 µm. The amount of residual carbon becomes much less. When further polished down to a depth of 100 µm from the top surface, similar phase compositions to those in (Ti_0.2_Nb_0.2_Ta_0.2_Hf_0.2_W_0.2_)C were observed.

**Fig. 7:**
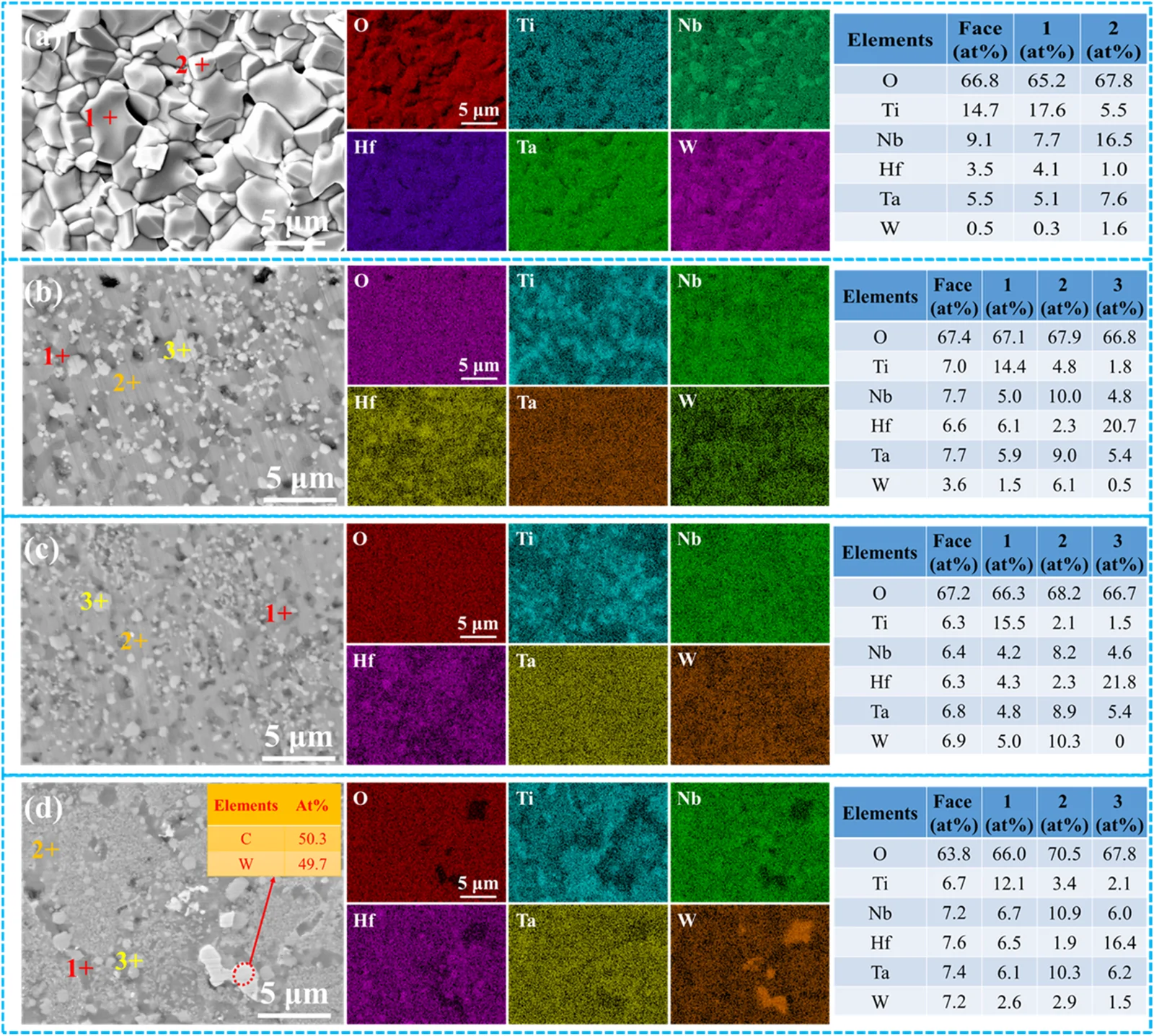
SEM images and corresponding EDS mappings of the four typical layers in the oxide scale of (Ti_0.2_Nb_0.2_Ta_0.2_Hf_0.2_W_0.2_)C_0.8_ (a) outermost layer; (b) second layer after polishing down to a depth of 30 µm from the top surface; and (c) third layer after polishing down to a depth of 60 µm from the top surface; (d) the fourth layer after polishing down to a depth of 80 µm from the top surface.

**Fig. 8:**
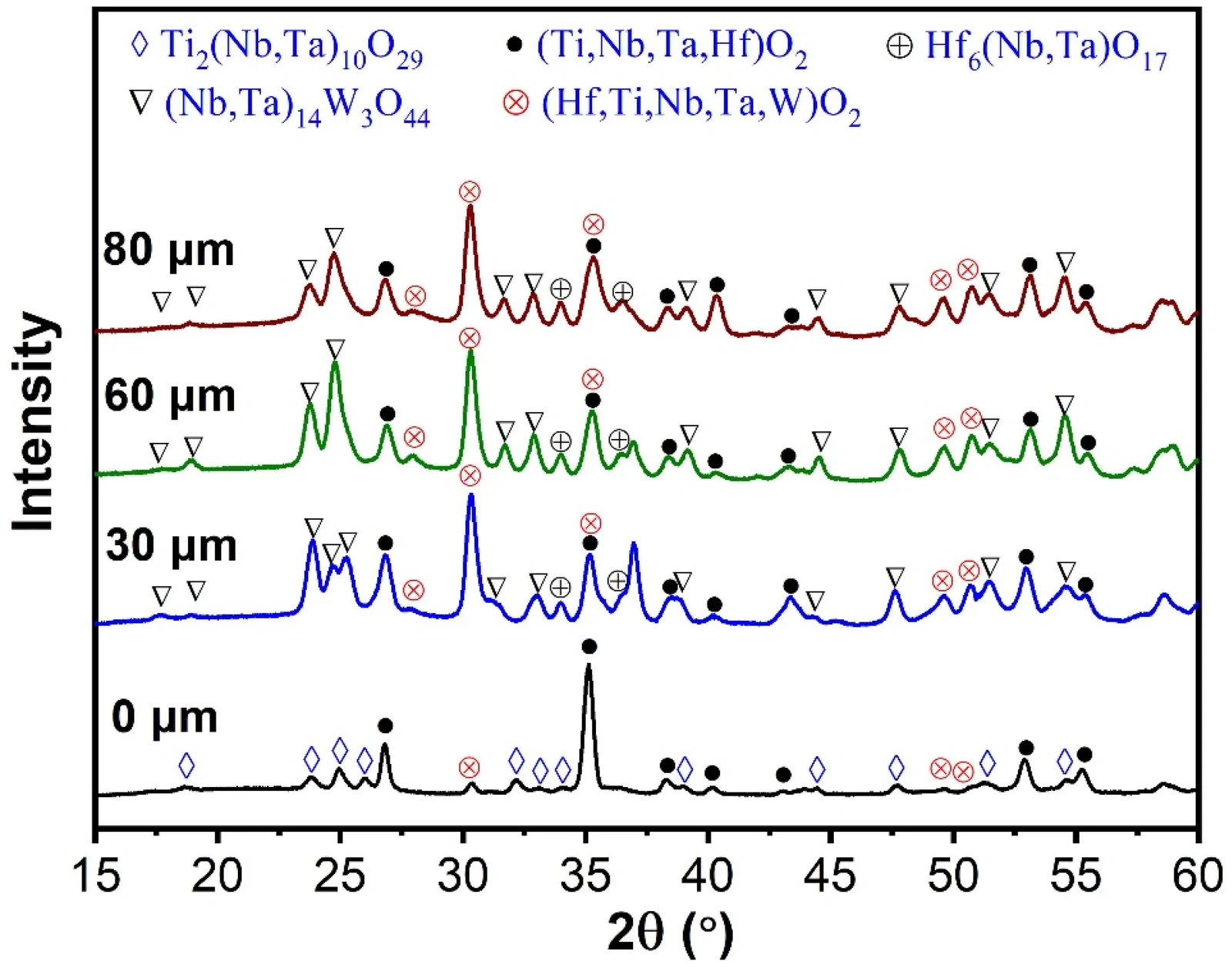
XRD patterns of different layers in the oxide scale of (Ti_0.2_Nb_0.2_Ta_0.2_Hf_0.2_W_0.2_)C_0.8_.

The SEM images and corresponding EDS mappings of the four typical layers in the oxide scale of (Ti_0.2_Nb_0.2_Ta_0.2_Hf_0.2_W_0.2_)C_0.8_ are displayed in Fig. 7. The first three layers resemble those of (Ti_0.2_Nb_0.2_Ta_0.2_Hf_0.2_W_0.2_)C_0.9_. The only difference is that the content of Nb-rich oxides Ti_2_(Nb,Ta)_10_O_29_ in the outermost surface becomes much higher. When polished down to a depth of 80 µm from the top surface, an additional layer with unaffected WC_1−*x*_ can be distinguished, which is consistent with the cross-sectional SEM morphologies of the oxidation layers in Fig. 2. The XRD patterns of the different layers of (Ti_0.2_Nb_0.2_Ta_0.2_Hf_0.2_W_0.2_)C_0.8_ are displayed in Fig. 8. The diffraction peaks of different layers resemble those of (Ti_0.2_Nb_0.2_Ta_0.2_Hf_0.2_W_0.2_)C_0.9_.

From the above experimental results, it can be summarized that the W-containing high-entropy (Ti_0.2_Nb_0.2_Ta_0.2_Hf_0.2_W_0.2_)C_*x*_ compositions exhibit high oxidation resistance in 1200 °C water vapor, and increasing the carbon vacancies further enhances the oxidation resistance. As is well known, the oxidation of transition-metal carbides proceeds through the inward diffusion of O and outward diffusion of transition-metal elements. At the initial oxidation stage, the carbon atoms in the FCC lattice structure are energetically favorable for substitution with oxygen atoms.^33^ The compositions with carbon vacancies are more favorable for both the inward diffusion of O and the outward diffusion of transition-metal elements. Consequently, (Ti_0.2_Nb_0.2_Ta_0.2_Hf_0.2_W_0.2_)C shows the lowest weight gain during the first 2 h. Oxygen is first dissolved in the carbides by forming oxycarbide, and when the oxycarbide is saturated with oxygen, further oxidation gives rise to oxides and residual carbon.^33–35^ Due to the presence of carbon vacancies, (Ti_0.2_Nb_0.2_Ta_0.2_Hf_0.2_W_0.2_)C_0.8_ shows dense oxide layers with much less residual carbon compared to (Ti_0.2_Nb_0.2_Ta_0.2_Hf_0.2_W_0.2_)C. Consequently, (Ti_0.2_Nb_0.2_Ta_0.2_Hf_0.2_W_0.2_)C shows accelerated oxidation after 2 h due to the presence of large amounts of residual carbon, which provides relatively large channels for the water vapor to pass through.

Due to the weaker ability of W to adsorb O atoms, W–C bonds are more difficult to oxidize than the other TM-C bonds.^27^ This can be confirmed by the unaffected WC_1−*x*_ grains in the inner oxidation layer of (Ti_0.2_Nb_0.2_Ta_0.2_Hf_0.2_W_0.2_)C_0.8_, which is consistent with the result in the oxidation of (Hf, Ta, Zr, W)C high-entropy carbides up to 3600 °C.^31^ This is also one of the major reasons for the higher oxidation resistance of the W-containing HECs. The oxidation process of (Ti_0.2_Nb_0.2_Ta_0.2_Hf_0.2_W_0.2_)C_*x*_ is also largely controlled by the outward diffusion of transition-metal elements, which is evidenced by the layered structures with compositional heterogeneity in the oxidation scales.^18,36^ W atoms show much lower diffusion ability, which can be validated by the much lower W content in the top two layers. The lower diffusion ability of W also contributes greatly to the enhanced oxidation resistance.

To further elucidate the fundamental mechanistic differences in the oxidation of high-entropy carbides between water vapor and air environments, a comparative analysis was conducted from two core dimensions: selective oxidation behavior and phase evolution pathways. Such a distinct oxidation behavior is experimentally supported by the comparison between the surface morphology and elemental distribution after oxidation in air at 1200 °C (Fig. S1) and after water vapor oxidation (Fig. 3). In air environments with sufficient oxygen partial pressure, the abundant oxygen supply diminishes the differences in the intrinsic oxidation resistance of each element, causing the metal elements in the matrix to undergo oxidation almost simultaneously. Consequently, the W element is distributed relatively uniformly throughout the oxide layer, and the oxidation products can directly form multi-component oxide solid solutions.^27^ Correspondingly, the phase evolution pathway during air oxidation is more direct, allowing the formation of stable complex oxide phases (such as (Zr,Hf)_6_(Nb,Ta)_2_O_17_) in a single step, without notable intermediate reaction products. In contrast, water vapor oxidation occurs under oxygen-limited conditions, where the required oxygen is continuously supplied solely through the dissociation of H_2_O molecules at the gas–solid interface. This low oxygen activity condition significantly amplifies the selective oxidation tendency among the elements, leading to pronounced elemental stratification and stepwise phase evolution during the oxidation process.

Due to the presence of W, the compositions of the mixed oxides in the oxidation layer differ from the HEC compositions without W. For HEC compositions without W, Ti tends to diffuse outward and the remaining Nb and Ta tend to form (Nb,Ta)_2_O_5_.^16,37–39^ It has been reported that the presence of Nb_2_O_5_ and Ta_2_O_5_ generally leads to cracks induced by thermal stress in the oxide layers, and the voids between the Nb–O and Ta–O octahedra form channels that facilitate oxygen diffusion.^16,37,38,40^ Consequently, (Nb,Ta)_2_O_5_ is characterized as a detrimental oxide concerning oxidation resistance performance.^38^ It has been reported that NbTa-depleted (Nb_0.125_Ta_0.125_Zr_0.375_Hf_0.375_)C exhibits superior antioxidant performance compared to the equiatomic (Nb,Ta,Zr,Hf)C, which is generally ascribed to the disappearance of (Nb,Ta)_2_O_5_ in the oxidation layer of (Nb_0.125_Ta_0.125_Zr_0.375_Hf_0.375_)C.^16^

Notably, the dynamic evolution of (Nb,Ta)_2_O_5_ under water vapor conditions has been directly verified in recent studies. Su *et al.* demonstrated that during the water vapor oxidation of the Cr/Ti-containing high-entropy ceramic (Ti,Zr,Nb,Ta,Cr)C, (Nb,Ta)_2_O_5_ initially forms the inner oxide sublayer.^18^ It is subsequently consumed *via* chemical reactions as the oxidation proceeds, yielding a larger and denser complex oxide phase TiO_2_-(Nb,Ta)_2_O_5_, which effectively inhibits the inward diffusion of H_2_O(g) even without forming cracks. Furthermore, the study by Tan *et al.* also confirms the formation and evolution behavior of (Nb,Ta)_2_O_5_ during the water vapor oxidation of high-entropy carbides.^17,19^

In this study, due to the presence of W, the EDS and XRD results indicate the formation of (Nb,Ta)_14_W_3_O_44_ instead of (Nb,Ta)_2_O_5_ in the oxide scale. This may be attributed to a possible reaction between (Nb,Ta)_2_O_5_ and WO_2_/WO_3_. Such a reaction is consistent with previous thermodynamic studies by Viccary *et al.*, who reported the formation of W_3_Nb_14_O_44_ from Nb_2_O_5_ and WO_3_ even at temperatures below 1000 °C.^41^ Meanwhile, the oxide scale appears dense and crack-free, which would be beneficial for suppressing the inward diffusion of water vapor. In summary, these findings demonstrate that the incorporation of W and the introduction of carbon vacancies synergistically enhance the oxidation resistance by refining the oxide scale microstructure and eliminating detrimental oxide phases.

**Fig. 9:**
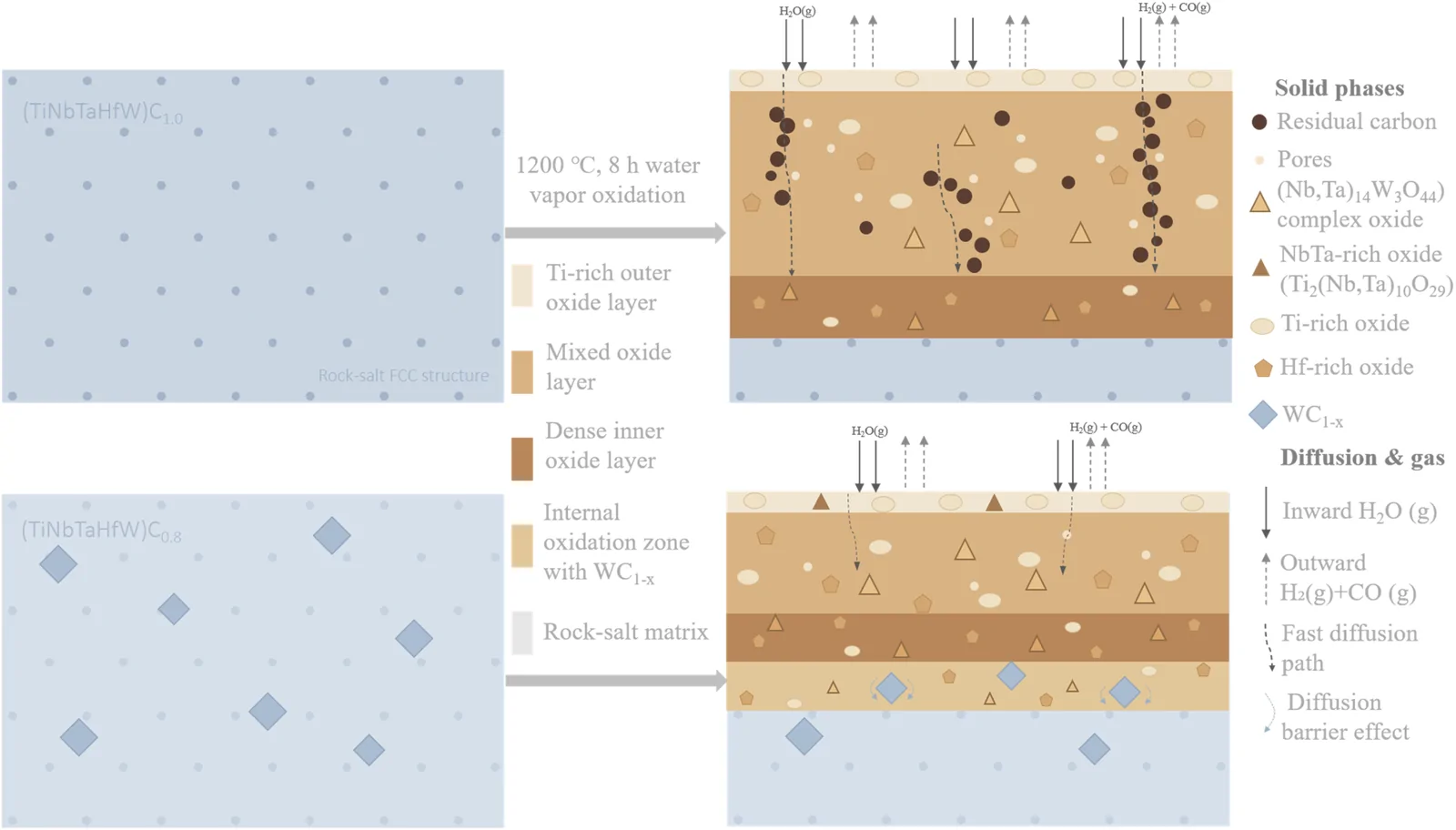
Schematic of the oxidation mechanisms for (Ti_0.2_Nb_0.2_Ta_0.2_Hf_0.2_W_0.2_)C_1.0_ (top) and (Ti_0.2_Nb_0.2_Ta_0.2_Hf_0.2_W_0.2_)C_0.8_ (bottom). Residual carbon in C_1.0_ forms continuous fast diffusion paths for water vapor, accelerating the oxidation process. In contrast, carbon vacancies in C_0.8_ reduce residual carbon, and the retained unoxidized WC_1−*x*_ second phases act as physical barriers to prolong diffusion paths, jointly improving the water vapor oxidation resistance.

To better illustrate the proposed oxidation mechanism, a schematic diagram comparing the (Ti_0.2_Nb_0.2_Ta_0.2_Hf_0.2_W_0.2_)C and (Ti_0.2_Nb_0.2_Ta_0.2_Hf_0.2_W_0.2_)C_0.8_ samples is presented in Fig. 9. As summarized in the diagram, the superior oxidation resistance of the (Ti_0.2_Nb_0.2_Ta_0.2_Hf_0.2_W_0.2_)C_0.8_ sample originates from the synergistic effects of: (i) reduced residual carbon in the oxide layer due to the presence of carbon vacancies; (ii) the low oxygen affinity and low diffusivity of W, which limits outward W migration; and (iii) the elimination of detrimental (Nb,Ta)_2_O_5_ through its reaction with WO_2_/WO_3_. These factors collectively result in a dense, crack-free oxide scale that effectively inhibits the inward diffusion of water vapor.

## Conclusions

The oxidation behavior of (Ti,Nb,Ta,Hf,W)C_*x*_ in high-temperature water vapor was systematically studied. (Ti,Nb,Ta,Hf,W)C_*x*_ show superior oxidation resistance compared to the well-studied (Ti,Zr,Nb,Ta,Hf)C, and the oxidation resistance increases with increasing carbon vacancies. The weaker O atom adsorbing ability and the lower diffusion ability of the W element are among the main reasons for the higher oxidation resistance. Additionally, the decrease in residual carbon in the oxidation layer with increasing carbon vacancies and the disappearance of the (Nb,Ta)_2_O_5_ phase effectively inhibit the inward diffusion of water vapor.

## Author contributions

Wei Liao: conceptualization, data curation, visualization, writing – original draft. Li Xu: methodology Jiashan Xie: investigation Xiaosong Zhou: project administration, supervision, writing – review and editing. Yongqiang Tan: validation, supervision, funding acquisition, writing – review and editing.

## Conflicts of interest

There are no conflicts to declare.

## Supplementary Material

RA-016-D6RA04394A-s001

## Data Availability

All data supporting this study are included within the article and its supplementary information (SI) files. Supplementary information is available. See DOI: https://doi.org/10.1039/d6ra04394a.

## References

[cit1] Fahrenholtz W. G., Hilmas G. E. (2017). Scr. Mater..

[cit2] Ni D., Cheng Y., Zhang J., Liu J.-X., Zou J., Chen B., Wu H., Li H., Dong S., Han J., Zhang X., Fu Q., Zhang G.-J. (2021). J. Adv. Ceram..

[cit3] Levine S. R., Opila E. J., Halbig M. C., Kiser J. D., Singh M., Salem J. A. (2002). J. Eur. Ceram. Soc..

[cit4] Katoh Y., Vasudevamurthy G., Nozawa T., Snead L. L. (2013). J. Nucl. Mater..

[cit5] Opeka M. M., Talmy I. G., Zaykoski J. A. (2004). J. Mater. Sci..

[cit6] Shimada S., Onuma T., Kiyono H., Desmaison M. (2006). J. Am. Ceram. Soc..

[cit7] Gasparrini C., Chater R. J., Horlait D., Vandeperre L., Lee W. E. (2018). J. Am. Ceram. Soc..

[cit8] Wei B., Wang D., Wang Y., Zhang H., Peng S., Xu C., Song G., Zhou Y. (2018). RSC Adv..

[cit9] Réjasse F., Trolliard G., Léchelle J., Rapaud O., Carles P., Grauby O., Khodja H. (2018). Acta Mater..

[cit10] Gong G., He Q., Wang Y., Yin X. (2026). Ceram. Int..

[cit11] Liu H., Sun Y., Zhang X., Liu B., Han L., Fu Q., Yin X., Li H. (2025). Nano Res..

[cit12] Zhang Y., Yin X., Li H. (2025). Matter.

[cit13] Ye B., Wen T., Liu D., Chu Y. (2019). Corros. Sci..

[cit14] Ye B., Wen T., Chu Y. (2020). J. Am. Ceram. Soc..

[cit15] Wang H., Wang S., Cao Y., Liu W., Wang Y. (2021). J. Mater. Sci. Technol..

[cit16] Li Y., He L., Pan H., Zhao S., Wu Z. (2025). Acta Mater..

[cit17] Tan Y., Chen C., Li S., Han X., Xue J., Liu T., Zhou X., Zhang H. (2020). J. Alloys Compd..

[cit18] Su W., Chen L., Huo S., Zhang W., Wang Y., Zhou Y. (2024). Corros. Sci..

[cit19] Tan Y., Teng Z., Jia P., Zhou X., Zhang H. (2021). Corros. Sci..

[cit20] Wang Y., Reece M. J. (2021). Scr. Mater..

[cit21] Backman L., Gild J., Luo J., Opila E. J. (2020). Acta Mater..

[cit22] Backman L., Gild J., Luo J., Opila E. J. (2020). Acta Mater..

[cit23] Yu D., Tan Y., Zhang H. (2021). Ceram. Int..

[cit24] Chen Z., Zhang W., Fu F., Li M., Zhang J., Ren L., Zhang F., Wang W., Fu Z. (2025). J. Mater. Res. Technol..

[cit25] Harrington T. J., Gild J., Sarker P., Toher C., Rost C. M., Dippo O. F., McElfresh C., Kaufmann K., Marin E., Borowski L., Hopkins P. E., Luo J., Curtarolo S., Brenner D. W., Vecchio K. S. (2019). Acta Mater..

[cit26] Liao W., Teng Z., Bao Y. (2025). Ceram. Int..

[cit27] Gong W., Yang Y., Li Z., Ye L., Cui H., Han W., Zhang P., Zhao T., Chen T. (2025). J. Eur. Ceram. Soc..

[cit28] Zhang W., Chen L., Shi Z., Lu W., Huo S., Wang Y., Zhou Y. (2024). J. Eur. Ceram. Soc..

[cit29] Zhu Y., Chai J., Wang Z., Shen T., Niu L., Li S., Jin P., Zhang H., Li J., Cui M. (2022). J. Eur. Ceram. Soc..

[cit30] Medved' D., Ivor M., Kovalčíková A., Múdra E., Csanádi T., Sedlák R., Ünsal H., Tatarko P., Tatarková M., Šajgalík P., Dusza J. (2022). Int. J. Appl. Ceram. Technol..

[cit31] Wen Z., Liu Y., Yang J., Chen Y., Fu Y., Zhuang L., Yu H., Chu Y. (2025). Adv. Mater..

[cit32] Tan Y., Liao W., Teng Z., Zhang H. (2023). J. Am. Ceram. Soc..

[cit33] Zhuang L., Wen Z., Liu Y., Yang J., Yu H., Chu Y. (2025). Adv. Mater..

[cit34] Shimada S. (2001). Solid State Ionics.

[cit35] Kiyono H., Shimada S., Sugawara K., Christensen A. (2003). Solid State Ionics.

[cit36] Tan Y., Liao W., Xia Y., Teng Z., Jia P., Zhou X., Zhang H. (2022). Corros. Sci..

[cit37] Peng Z., Sun W., Xiong X., Zhang H., Guo F., Li J. (2021). Corros. Sci..

[cit38] Li Y., Wang Y., Lin N., Zhao S., Wu Z. (2025). Corros. Sci..

[cit39] Wang Y., Wang X., Zhang B., Zhang H., Reece M. J. (2024). Ceram. Int..

[cit40] Nakamura R., Toda T., Tsukui S., Tane M., Ishimaru M., Suzuki T., Nakajima H. (2014). J. Appl. Phys..

[cit41] Viccary M. W., Tilley R. J. D. (1993). J. Solid State Chem..

