# Supplementary material for: Water vapor oxidation behavior of high-entropy (Ti,Nb,Ta,Hf,W)Cx at 1200 °C: the role of carbon vacancies
Source: RSC Adv. 2026 Aug 17;16(41):48472–81. doi: 10.1039/d6ra04394a (PMC13480447; doi:10.1039/d6ra04394a)
Supplement: Supplementary file 1 [file RA-016-D6RA04394A-s001.pdf]

Supplementary Information (SI) for RSC Advances.
This journal is © The Royal Society of Chemistry 2026

Water vapor oxidation behaviors of high-entropy (Ti,Nb,Ta,Hf,W)C_x at 1200 °C

Wei Liao[a,b,*], Li Xu[b], Jiashan Xie[b], Xiaosong Zhou[b,*], and Yongqiang Tan[b]

[a] *Department of Materials Science and Engineering, University of Science and Technology of China, Hefei, 230026, China*

[b] *Institute of Nuclear Physics and Chemistry, China Academy of Engineering Physics, Mianyang, 621900, China*

*Corresponding authors:
Liaowei96@mail.ustc.edu.cn,
zlxs77@126.com

Figure captions

Fig. S1 Schematic flow chart of the reactive SPS sintering process for the high-entropy carbides.

Fig. S2 Surface SEM morphology and corresponding EDS spectrum of $(Ti_{0.2}Nb_{0.2}Ta_{0.2}Hf_{0.2}W_{0.2})C$ after isothermal oxidation at 1200 °C in air for 1 h.

Figure S1

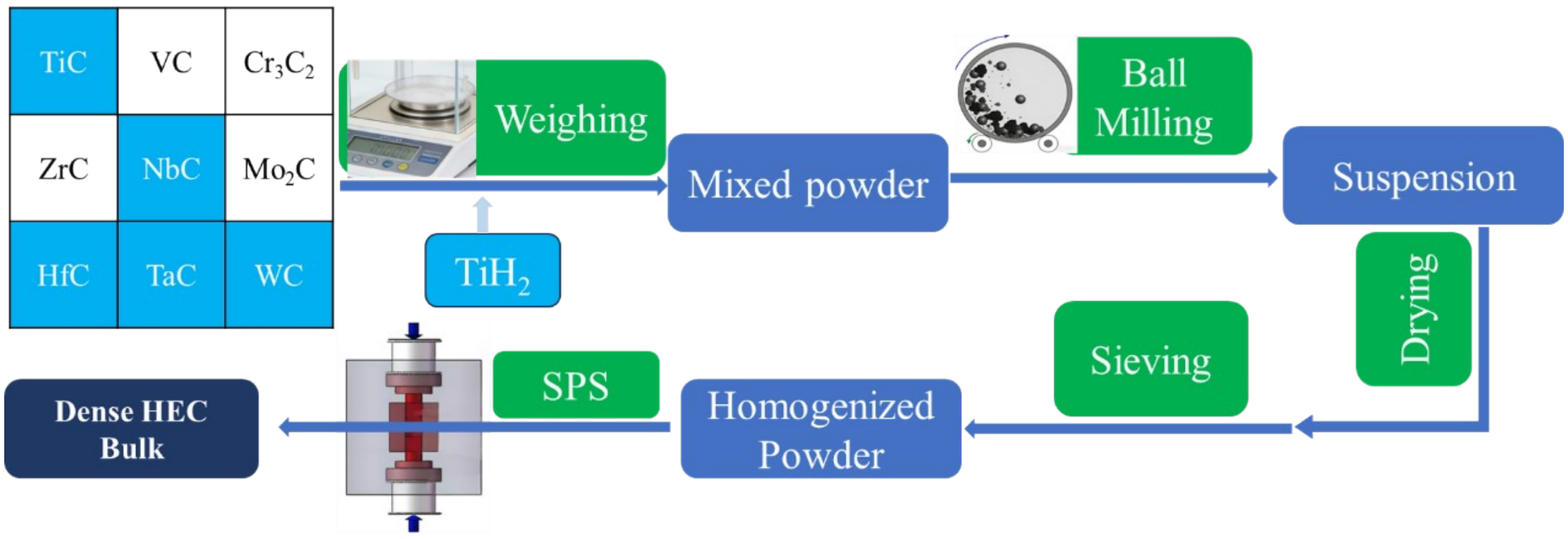
TiC
VC
Cr3C2
ZrC
NbC
Mo2C
HfC
TaC
WC
Weighing
TiH2
Mixed powder
Ball Milling
Suspension
Drying
Sieving
Homogenized Powder
SPS
Dense HEC Bulk

Figure S2

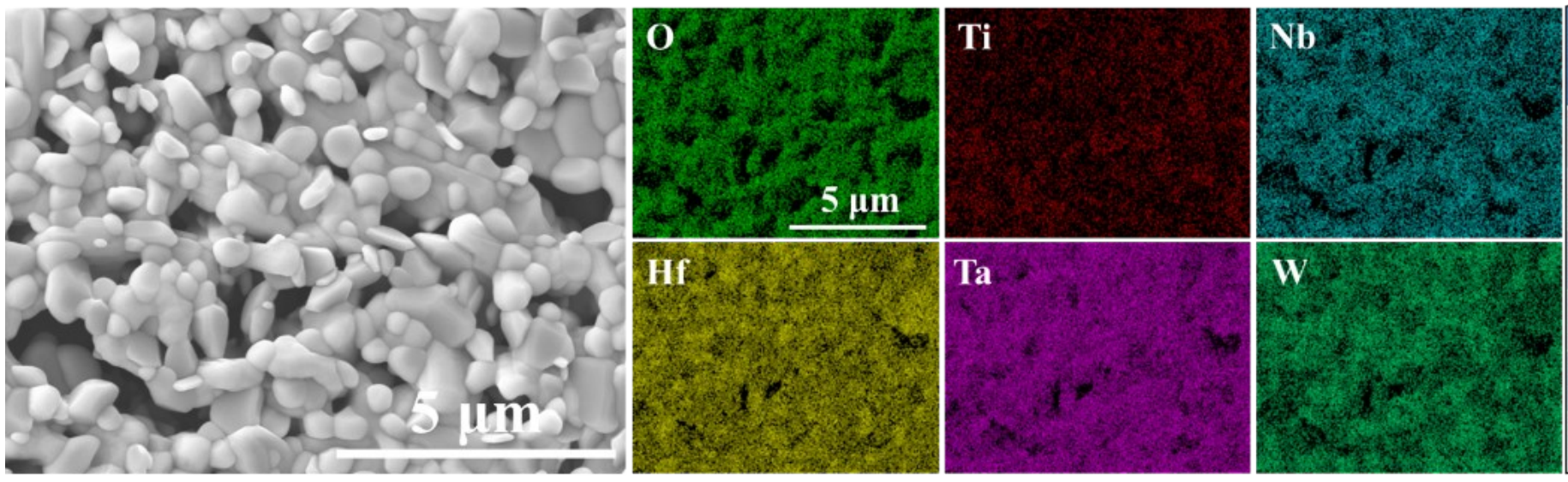

Elements	Face(at%)
O	65.4
Ti	10.3
Nb	4.9
Hf	10.1
Ta	6.7
W	2.6
